# Semantic Processing of Arabic Numbers Across Tasks

**DOI:** 10.1111/psyp.70082

**Published:** 2025-06-03

**Authors:** Will Deng, Danielle S. Dickson, Kara D. Federmeier

**Affiliations:** ^1^ Department of Psychology University of Illinois Champaign Illinois United States; ^2^ Program in Neuroscience University of Illinois Urbana Illinois United States; ^3^ Beckman Institute for Advanced Science and Technology University of Illinois Urbana Illinois United States

**Keywords:** ERPs, knowledge access, N400, numerical cognition

## Abstract

Numbers are used in a variety of ways and in different contexts—as labels, markers of ordinality, or indicators of quantity, in addresses and phone numbers and in mathematical equations. This raises the question of whether knowledge access from numbers involves similar or distinct mechanisms across these uses and how it compares to accessing knowledge from words and pictures. To investigate this, we presented double‐digit numbers in three tasks designed to target different types of information: a matching task requiring access only to number form, a divisor task situating numbers in the context of basic arithmetic, and a quantifier task using numbers to represent everyday quantities. We measured event‐related potentials (ERPs), focusing on the N400 component, which has been linked to access from long‐term semantic memory, and looking at the impact of repetition as an implicit probe of facilitated knowledge retrieval. Our results revealed reliable N400 repetition effects for numbers across all tasks, suggesting that numbers are linked to associated representations of numerosity in a relatively automatic manner, using similar mechanisms as the access of semantics from words and pictures. However, consistent with claims that representations of numerosity involve different brain networks compared to general semantics, the scalp distribution of the N400 repetition effect for numbers, which was consistent across our three tasks, differed from that to words in the present experiment and from that observed in prior work using numbers to access general semantics.

## Abstract 2

Using event‐related potentials (ERPs) in a repetition paradigm, we found that accessing numerosity information from Arabic numbers produces consistent N400 effects across tasks involving simple form matching, arithmetic, and quantity judgments, indicating relatively automatic processing. However, differences in N400 scalp distributions between numbers and words suggest that distinct brain networks are involved in representing numerosity versus general semantics.

Humans have the remarkable ability to associate arbitrary symbols with stored knowledge. This kind of arbitrary mapping is crucial for understanding and using both words and numbers; although, of course, the specific types of information conferred by numbers and words can differ. Whereas words are used to evoke mental representations of a wide array of knowledge types, from concrete objects to actions to various abstract concepts and properties, numbers are often used to convey quantities and ordinal information; although they can also have broader uses (e.g., as identifiers or labels, such as with serial numbers or on a sports jersey). A question of longstanding interest is whether knowledge access from words and numbers is mechanistically similar or different (Lachmair et al. 2014; Pimperton and Nation 2010). On the one hand, the brain areas that have been linked to representations of word semantics and numerical information are not identical. Representations of numeric information have been localized to regions in the intraparietal sulcus (Ansari et al. 2006; Dehaene et al. 1999); whereas temporal areas have been argued to be critical for representations of word semantics (Binder et al. 2009; Friederici et al. 2017). On the other hand, the process of accessing semantics from various kinds of stimuli has been broadly associated with the same type of neural activity, as measured in the N400 component of the event‐related potential (ERP).

The N400 is a negative‐going component that peaks around 400 ms after stimulus onset. It is postulated to reflect initial activation states in long‐term semantic memory, with larger (more negative) N400s observed to stimuli that elicit more new activity in semantic memory. There are a number of well‐established paradigms that are used to elicit reliable N400 effects (Federmeier 2022). An extensive literature using word stimuli has shown that N400 amplitudes are reduced for stimuli that are semantically related to prior context information (e.g., semantic priming, fit to a sentence; see review in Kutas and Federmeier 2011). Similar context‐based N400 effects have also been observed for meaningful non‐verbal stimuli like faces, pictures, and environmental sounds (Ganis et al. 1996; Olivares et al. 1999; Van Petten and Rheinfelder 1995). N400 amplitude reductions are also robustly observed when stimuli are simply repeated after a short time (Rugg et al. 1994; Van Petten et al. 1991), and these effects have also been attested for various types of meaningful stimuli, with the size of the repetition effect scaling with the number of repetitions and the spacing between them, among other factors (e.g., Besson and Kutas 1993; Voss and Paller 2006; Woodruff et al. 2006). Thus, the N400 has been argued to reflect a general mechanism that links perceptual inputs of all kinds to information stored in long‐term semantic memory and that is facilitated when some of the information normally evoked by the stimulus has already been made available through context or recent exposure (Federmeier et al. 2016; Federmeier 2022).

If the semantic access involved in number processing is similar to that for words, we would expect to see an N400 effect for numbers. Indeed, this seems to be the case when numbers are being used to access the kind of semantic information that words might also be used to access. Dickson and Federmeier (2018) presented participants with double‐digit numbers, which were each repeated after a few trials. To encourage participants to link these numbers to semantics broadly, participants were asked to indicate their “familiarity” with each number and told to judge familiarity by considering things like “apartment numbers, people's ages, dates and years, and athlete jersey numbers.” The results revealed an N400 repetition effect, which obtained whether or not the number was rated as familiar, with a reduced N400 for the second presentation. Both the time course (peaking a little before 400 ms) and scalp topography (centroparietal maximum) of the N400 repetition effect to numbers in this paradigm was similar to that previously established for visual words. This pattern suggests that, as for words, initial exposure to a number can help facilitate access to semantic knowledge linked to that number at the second presentation, at least under conditions in which numbers are connected to general knowledge and experience.

However, numbers are also used for different purposes, associated with the access of different types of information. One common use of numbers is in arithmetic expressions. Initial ERP work looking at the responses to numbers in the context of simple math problems (e.g., “4 × 6 =”) described a pattern wherein correct answers (24) compared to incorrect answers (26) elicited more positive responses beginning around 300 ms after presentation of the solution (Niedeggen and Rösler 1999; Niedeggen et al. 1999); this pattern was interpreted as an N400 effect, similar to effect patterns seen for words that fit versus are anomalous in a sentence frame (Kutas and Hillyard 1980). However, another ERP component that can be observed in the same time frame of the N400 and with a broadly similar distribution is the P3b, a posteriorly‐ distributed, positive‐going potential that is sensitive to probability or expectedness (e.g., Duncan‐Johnson and Donchin 1977) and that is enhanced (more positive amplitudes) for target stimuli in detection tasks (Polish 2012). Given that these components are potentially confusable, questions began to arise about whether the effect observed for solutions of arithmetic expressions was best described as an N400 or a P3b. Later work established that, for adults with well‐practiced arithmetic skills, correct answers to written arithmetic problems tend to be treated like targets, eliciting a P3b (Dickson et al. 2018; Jasinski and Coch 2012; Wood et al. 2023). Thus, it seems likely that earlier work reporting N400 effects in the context of arithmetic expressions were actually measuring a difference arising from a larger (more positive) P3b—rather than a smaller (less negative) N400—to correct solutions.

The fact that Arabic numbers completing arithmetic expressions elicit P3b effects but, at least for adults^1^, not N400 effects raises the possibility that when numbers are used to designate quantities (as is very typical) they are not processed the same way as words and pictures. It is already known that not all forms of expectancy in familiar and potentially meaningful sequences are associated with N400 effects. For example, responses to deviant notes in familiar musical melodies elicit P3b‐like effects, not N400s (Besson and Macar 1987; Paller et al. 1992). Perhaps the information conveyed by well‐learned arithmetic expressions is similar to the case of learned musical patterns, where the limited semantic message is insufficient to elicit the kind of N400 effects observed for sentences, picture sequences, and movie clips. Alternatively, it is possible that numbers do elicit N400 effects, even when used to convey quantity‐based or ordinal information, if probed outside of over‐learned sequences like simple arithmetic expressions, where P3b effects dominate the response. To assess this, we here developed a repetition‐based paradigm in which N400 effects to numbers can be generated in the absence of an overlapping P3b. In this design, the critical, repeating stimuli are presented before any decisions can be made about the numbers, and the decision‐related information is provided as a delayed probe item. Through this method, rather than measuring the amount of facilitation an item receives as a function of a prior arithmetic expression, we will instead be simply measuring the amount of facilitation an item receives as a function of its own prior presentation earlier in a list—that is, using a standard repetition paradigm to elicit N400 effects.

Repetition paradigms have been widely used to examine the semantic processing of a potentially meaningful item, with reductions in N400 reflecting conceptual priming (e.g., Voss and Paller 2006; Voss et al. 2010; Voss and Federmeier 2011). The amount of conceptual processing has been found to influence the size of the effect. For example, although N400 priming effects are often small or absent for meaningless, illegal strings (e.g., “TXQ”), if participants are encouraged to entertain the possibility that these items are meaningful by judging whether they are a familiar acronym, illegal strings can manifest N400 repetition effects similar to other, meaningful strings of letters (e.g., words and acronyms; Laszlo et al. 2011). Similarly, although N400 effects are difficult to find when people are processing melodies, it is possible to obtain them through cross‐modal priming studies with language stimuli and other sophisticated manipulations that tap into richer aspects of the meaning of musical elements (Daltrozzo and Schön 2009; Koelsch et al. 2004). As already described, numbers can elicit N400 effects when the task explicitly encourages broad semantic associations (Dickson and Federmeier 2018). Here, we extend this paradigm to investigate whether numbers will also elicit N400 effects in other contexts.

We used three tasks, each of which recruits different types of engagement with (the same) numbers' potential associations. All tasks involve the presentation of an Arabic number, followed by a probe stimulus to which the participants respond; the numbers are then repeated across the course of the task. Critically, what is measured in these tasks is always the response to the first and second presentation of the pre‐probe numbers. This allows us to directly measure how the size and morphology of the repetition effect for the exact same physical stimuli is influenced by the processing demands of each task, and thereby assess to what extent numbers are semantically primed by prior exposure across these different tasks. Because participants do not know what the probe stimulus will be when they encounter each number, the numbers are never targets and thus would not be expected to elicit a P3b.

In the “matching” task, participants view a double‐digit number and later verify the presence or absence of a probe digit in the number (e.g., they see 35 followed by either 3 or 6 and press a button to indicate if these single digit probe numbers were present in the first number). This encourages participants to see numbers as objects with subparts but does not require them to access additional numerosity‐based or conceptual information. The critical question this task asks is whether numbers are obligatorily processed for their conceptual associations. In similar paradigms using words, N400 repetition effects have been observed, showing that meaning extraction for words is relatively automatic (Holcomb 1988; Küper and Heil 2009; Olichney et al. 2000). However, for other items that are less inherently meaningful than words (e.g., ambiguous and novel line drawings), simply detecting visual features does not always result in N400 effects; instead, such effects are only obtained when participants are encouraged to think about these stimuli as potentially meaningful (Voss et al. 2010). Arabic numbers can elicit N400 effects when they are used as explicit cues for meanings (Dickson et al. 2018). However, it is unknown whether similar effects will occur when people are not explicitly directed to view them that way.

In the “divisor task”, participants view a number and later verify whether a probe number is one of its factors (e.g., seeing “14”, participants would confirm probe items of “1”, “14”, “2”, and “7”). If retrieving associated arithmetic facts activates a semantic network for the number itself, then one would expect numbers in this task to elicit an N400 repetition effect. This task thus provides a means of examining number processing in the context of simple arithmetic without contamination from a potentially overlapping P3b, since we are looking at effects on the number before a solution—and hence potential “target” stimulus—has been presented.

Finally, in the “quantifier task” we attempted to tap more deeply into representations of numerosity, using numbers as cues for real‐world quantities. We typically assume that the critical information being accessed from numbers is their numerosity, but the results from simple arithmetic problems with adults emphasize that, even in ostensibly mathematical contexts, numbers may trigger the retrieval of memorized sequences (e.g., multiplication facts) that do not necessarily make contact with deeper representations of numerical quantity (Dickson et al. 2018; Grenier et al. 2020). Under the assumption that most people's natural experience with numbers outside the realm of the classroom is in the context of measuring and counting, the quantifier task asks people to read numerical expressions (e.g., “85” followed by the quantifier “yards”) and judge them for plausibility (how well the quantity and the label go together). This task is thus aimed at tapping into people's conceptual knowledge of the size/magnitude conveyed by the numeral in a way that is less likely to be subserved by rote memory.

The main question being addressed by this study is under what contexts (basic perception, arithmetic expression, cues to quantities) are numbers processed more like semantic cues, such that they elicit N400 repetition effects. In general, the approach of using single‐item presentations with repetitions of identical or related primes has been critical for honing our understanding of the temporal dynamics of extracting various properties (such as semantics) from a diverse group of stimuli across modalities, such as visual and auditory words (e.g., Rugg et al. 1993; Joyce et al. 1999; Holcomb and Grainger 2006, 2007), faces (e.g., Schweinberger et al. 2002; Schweinberger et al. 2004), dynamic gestures (Corina et al. 2011; Wu and Coulson 2005), environmental sounds (Schirmer et al. 2011), and pictures of objects (McPherson and Holcomb 1999; Schendan and Kutas 2003, 2007; Eddy et al. 2006), so this study will support and extend the existing broader literature of how different classes of stimuli are assessed and processed for meaning.

## 1 Methods

### 1.1 Participant

A total of 30 participants (15 self‐identifying as women and 15 as men) were included in the final analysis. All participants were native monolingual English speakers with normal or corrected‐to‐normal vision. Their average age was 21 years (range: 18–29). None reported early exposure to a second language, a history of brain trauma, or current use of psychoactive medication. They were all right‐handed, with a mean laterality quotient on the Edinburgh handedness inventory (Oldfield 1971) of 0.81 (range: 0.43–1.00, with 1.0 indicating strongly right‐handed and −1.0 strongly left‐handed); 12 reported having a left‐handed biological family member. To obtain the final sample of 30 participants, 33 individuals were initially recruited. Three were excluded prior to analysis due to failure or inability to comply with instructions (e.g., falling asleep or excessive blinking during critical trials). All participants provided informed consent in accordance with the guidelines of the Institutional Review Board at the University of Illinois.

### 1.2 Materials

#### 1.2.1 Critical Stimuli for All Tasks

All possible two‐digit numbers were included as critical stimuli, while filler items consisted of either single‐digit (1–9) or three‐digit numbers (100–116). Thus, from the participant's perspective, the full set of stimuli ranged from 1 to 116. Each critical number was repeated following a lag of two or three intervening numbers. Filler items were used to build the lag structure and did not repeat. The repetitions were not emphasized and bore no relevance to the task. To prevent priming effects due to partial numerical overlap, consecutive numbers with matching initial digits (e.g., 90 followed by 93) were avoided, and sequential matches of the second digit were infrequent. The same stimulus set was previously employed in Dickson and Federmeier (2018).

Three distinct stimulus orders and repetition structures were created. A number with two intervening items in one list could have three in another, and numbers appearing early in one version might be positioned later in a different one. This design allowed each participant to complete all three tasks and minimized the potential influence of list‐specific properties. Task order was counterbalanced across participants.

#### 1.2.2 Probe Stimuli for Matching and Divisor Tasks

In the Matching and Divisor tasks, critical numbers were followed by a single‐digit probe. To prioritize having the correct response be evenly distributed between YES/NO, the distribution of these probes differed across these tasks (i.e., there were a disproportionate number of probes of the digit 2 in the Divisor task). In the Matching task, the probe query required attention to have been paid to any one of the digits in the critical item in an unpredictable manner. Probes for correct answers were distributed evenly between the first digit and the second digit.

#### 1.2.3 Probe Stimuli for the Quantifier Task

In the Quantifier task, the critical numbers were followed by a probe quantifier word. These words included basic units of measurement (e.g., inches, gigabytes), as well as some collective nouns (used in an incomplete expression fragment, e.g., “pieces of…”), arrangement classifiers (“rows of…”) and varietal classifiers (“stages of…”, “types of…”).

In order to select quantifier words that were best associated with measurements and numbers, 105 English‐speaking participants from the United States were recruited to participate in a norming study through Amazon Mechanical Turk. These participants consented and were monetarily compensated in accordance with University of Illinois IRB policies. In the norming study, participants were given a sliding ruler that spanned 0–100, and their assignment was to select a number that felt “most natural” completing the expression or word they were provided with. For example, they saw, “—calories” and selected the number that they most naturally fit with the given measurement term. Critically, there was also a “No number feels natural” option, which was used to determine whether or not a given term would be sensible to our participants in the ERP experiment.

Data from 101 of 105 participants were usable; a few participants either skipped too many responses or picked “No number feels natural” for more than half the items. There were 100 potential words for measurement tested for viability. The threshold for exclusion was if five or more participants reported “No number feels natural” for a given word. This process removed 53 words from the set, leaving 47 to be used in the study.

Of the 47 quantifiers included, there was variability in the numbers that were selected to be natural‐sounding with them. The smallest average response was for “teaspoons” (mean: 6.30, mode: 2.0) and the largest average response was “calories” (mean: 87.95, mode: 100.0). The average of all responses was around 30 (29.19), and the majority of responses were below 50^2^. The 47 probe words were matched up with the 206 numerical items by repeating each probe 4–5 times (average: 4.4), with a buffer of at least 15 intervening items before a given probe word would be used again. Because the words were repeated, we were able to also directly compare N400 repetition effects to numbers and words in a within‐subject design.

### 1.3 Procedure

#### 1.3.1 General

In all tasks, the timing of items followed the same format. Prior to the critical stimulus, a series of red “~~~~” was presented to alert the participant that a number was coming up. To reduce the temporal predictability of stimulus presentation (and thereby reduce ERP contamination from anticipatory slow waves), this cue remained on the screen for a random period of time within the range of 400 and 600 ms. The critical stimulus was on the screen for 500 ms, followed by 800 ms of a blank screen. The probe query stimulus was then displayed for 500 ms, after which the screen went blank. Participants could respond as soon as they were able; speed was not emphasized. After a response was registered, a series of white “~~~~” was presented for 1500 ms, and participants were instructed that they could use this time to blink and move their eyes. The next red “~~~~” directly followed this period. A small square was presented continuously in the center of the screen just below the stimuli to remind participants where to fixate.

There were periodic self‐paced breaks in which yellow “~~~~” were presented after a response; this indicated that the participants could take a break until they were ready to do more trials. During each of the three tasks across the experiment, there were also two experimenter‐controlled breaks: one after an initial small batch of trials to check in on the participants' understanding of that task, and one roughly halfway through. Lists were structured to ensure that these breaks did not come in between repetitions.

Procedures that were unique to each task follow. Examples of primary task procedures are shown in Table 1.

**TABLE 1:** Examples of experimental stimuli in the three tasks.

Task	Critical number	Probe	Decision
Matching	12	2 (Yes) 6 (No)	If probe is one of the digits in the critical number
Divisor	36	6 (Yes) 8 (No)	If probe is a factor of the critical number
Quantifier	49	Gigabytes (Yes) Teaspoons (No)	If the probe is a common measurement associated with the critical number

*Note:* The correct response for each probe is indicated in parentheses.

#### 1.3.2 Matching Task

After reading the critical initial number, participants were probed with a second (single digit) number. They responded YES/NO to this number depending on whether that probe number was one of the digits in the first number. On trials in which the initial (filler) number was a single digit, participants were just answering whether the number repeated. More often, the first number was a double‐ or triple‐digit number whose parts or identity needed to be held in memory.

#### 1.3.3 Divisor Task

After reading the critical initial number, participants were probed with a second (single digit) number. They responded YES/NO to this number depending on whether the probe was a factor of the first number. Participants were specifically instructed to assess if they could “take the first number, divide it by the second number, and get a whole number”. They were further instructed that they did not need to know what the answer was, only whether or not it was a whole number.

#### 1.3.4 Quantifier Task

After reading the critical initial number, participants were probed with a quantifier word or expression that is typically used for measuring or counting items (as indicated by the prior norming study). Their instructions were to judge whether or not the previous number “seems typical for a given type of measurement” and to “decide if you think this is a common or typical pairing.” They were given examples of both rarer and more normal pairings, and it was further explained that there were no right answers because their judgments would depend on their own experience and familiarity with how numbers are used to express distances, sizes, amounts, and other measurements. After presentation of the word, participants responded either “reasonably common” or “reasonably uncommon”.

### 1.4 EEG Recording

Continuous EEG data were collected using 26 evenly spaced silver/silver‐chloride electrodes embedded in an elastic cap (Easycap GmbH). Electrode impedances were maintained below 5 kΩ. During recording, signals were referenced online to the left mastoid and subsequently re‐referenced offline to the average of the left and right mastoids. A separate electrode positioned at the frontal midline served as the ground. Vertical electrooculogram (EOG) activity was recorded via an electrode placed beneath the eye on the infraorbital ridge, while horizontal EOG was captured using electrodes positioned at the outer canthi of both eyes. The EEG signal was digitized at a sampling rate of 250 Hz and amplified through Sensorium EPA‐6 amplifiers using an analog bandpass filter from 0.02 to 100 Hz.

Raw EEG traces were evaluated for artifact contamination on a trial‐by‐trial basis, and artifact rejection criteria were determined independently for each participant through condition‐blind visual inspection. Trials were excluded if they contained eye blinks, saccades, excessive muscle activity, large signal drifts, or channel blocking. Following artifact rejection, the number of trials contributing to critical bins averaged 82 per participant (range: 55–90). For each trial, data were segmented into epochs spanning from 100 ms before to 900 ms after item onset, and the 100 ms pre‐stimulus interval served as the baseline. Condition‐based ERPs for each subject were extracted using the Event‐Related Potential Software System (developed by Jon Hansen). Mean amplitudes were measured after applying a digital bandpass filter of 0.1–20 Hz. N400 responses were quantified within a predefined window of 350–450 ms post‐stimulus, centered around the typical N400 peak.

Because the distribution of N400 effects elicited by Arabic numbers is not yet well established, electrode selection for evaluation of the task effect was informed by the results of a distributional analysis. Specifically, an initial distributional analysis was used to empirically select an ROI that can be used to probe for a task effect on the presence/size of the repetition effect. The initial analysis did not include task as a variable so as not to bias the later analysis. The interactions between the repetition effect and scalp distributional factors were used as an objective way to narrow down the ROI in order to provide better power for analyzing the task effect.

## 2 Results

### 2.1 Behavioral Data

Response accuracy for probe items was high in both the Matching Task (98.8%) and the Divisor Task (93.4%). In the Quantifier Task, where there were no right and wrong answers, the average percent of numerical expression endorsement was 54.7% (lowest endorsement rate: 22.3%; highest endorsement rate: 81%).

### 2.2 Event‐Related Potentials

**FIGURE 1:**
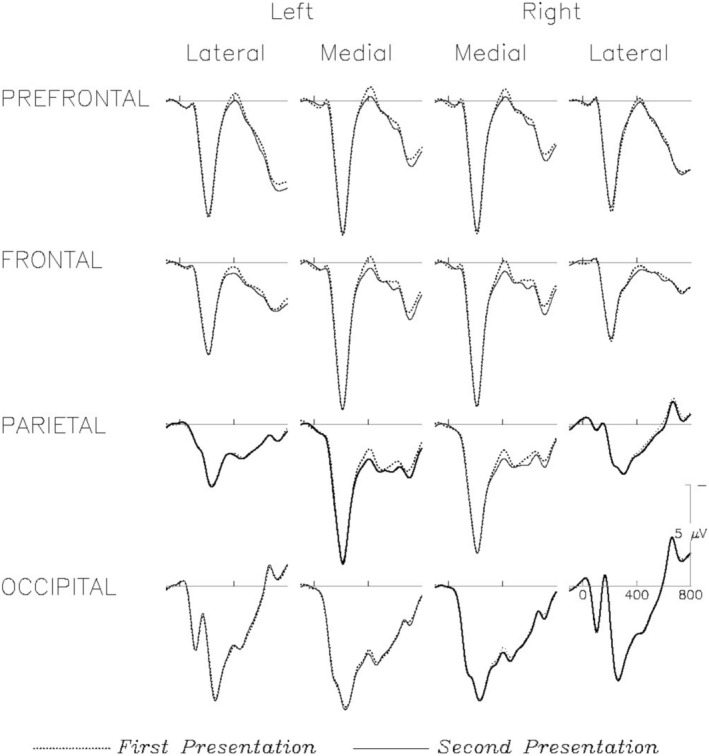
Shown are overall grand average ERPs to numbers, measured at the onset of their first (dotted lines) and second (solid lines) presentations. The depicted channels are those used in the distributional analysis. Negative voltage is plotted up. Amplitude reductions from the first to second presentation can be seen in the time window around 400 ms over medial prefrontal, frontal, and parietal channels, consistent with N400 repetition effects.

Overall (across task) responses to the first and second presentations of numbers over prefrontal, frontal, centro‐parietal, and occipital electrodes are shown in Figure 1. Following visual sensory potentials, responses to numbers were characterized by a negative‐going component peaking around 400 ms (N400), which was reduced for second presentations compared to first.

### 2.3 N400 Scalp Distribution to Arabic Numerals

To first assess the scalp distribution of N400 repetition effects elicited by numbers, we conducted an omnibus ANOVA using 16 channels distributed across the entire head. With this analysis, we obtain a statistically conservative assessment of the basic presence of an N400 repetition effect for Arabic numbers (across all tasks), and we then use the resulting topographic information to objectively select channels that are sensitive to number repetition effects for our planned examination of task effects. The analysis included two levels of repetition (first and second presentations), along with three distributional factors: two levels of hemisphere (left and right), two levels of laterality (lateral and medial), and four levels of anteriority (prefrontal, frontal, central, and occipital). There was a main effect of repetition (*F*(1, 29) = 8.15, *p* = 0.008), showing that there is indeed a repetition effect in the N400 time window for Arabic numbers, measured independent of task demands and across the entire scalp. The effect of repetition did not interact with hemisphere (*F*(1, 29) = 0.10, *p* = 0.751), but the interaction was significant for laterality (*F*(1, 29) = 7.15, *p* = 0.012) and marginally significant for anteriority (*F*(3, 87) = 2.63, *p* = 0.055). None of the higher order interactions between repetition and scalp distributional factors were significant^3^. Repetition effects were larger over medial sites (0.36 μV, SD = 0.78) compared to lateral sites (0.16 μV, SD = 0.68), and were larger over prefrontal (0.33 μV, SD = 0.81), frontal (0.39 μV, SD = 0.71), and central (0.25 μV, SD = 0.67) sites compared to occipital sites (0.07 μV, SD = 0.73). Given the outcome of the distributional analysis, for characterization of task effects, we selected a six‐electrode ROI consisting of electrodes over medial central, frontal, and prefrontal sites (LMPf, RMPf, LMFr, RMFr, LMCe, RMCe).

#### 2.3.1 N400 Task Effects for Numbers

**FIGURE 2:**
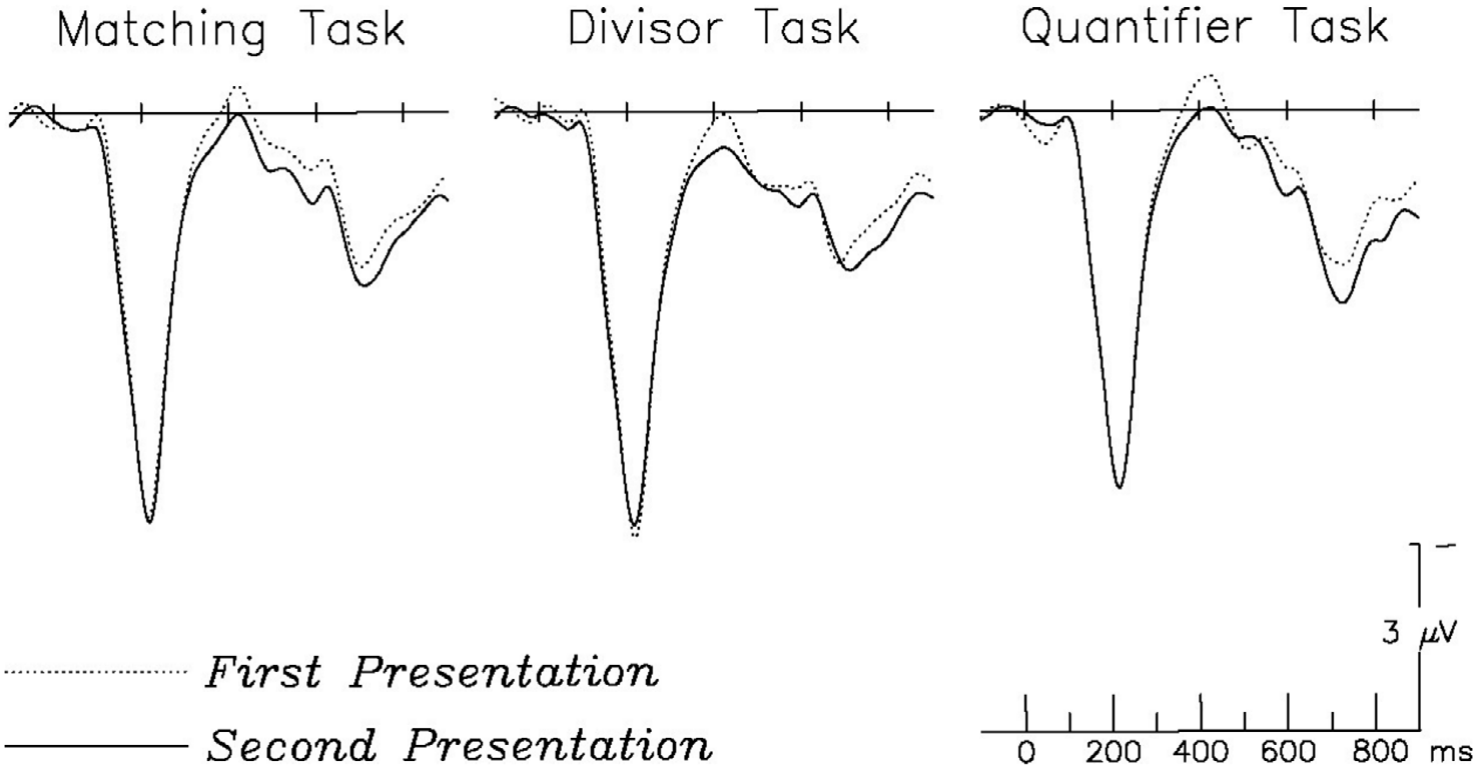
Shown are grand average ERPs for first (dotted lines) and second (solid lines) presentations of numbers in each task, plotted at a representative left medial frontal channel. Negative voltage is plotted up. N400 repetition effects across all three tasks can be observed as a reduction in amplitude from first to second presentations around the 400 ms time window.

**FIGURE 3:**
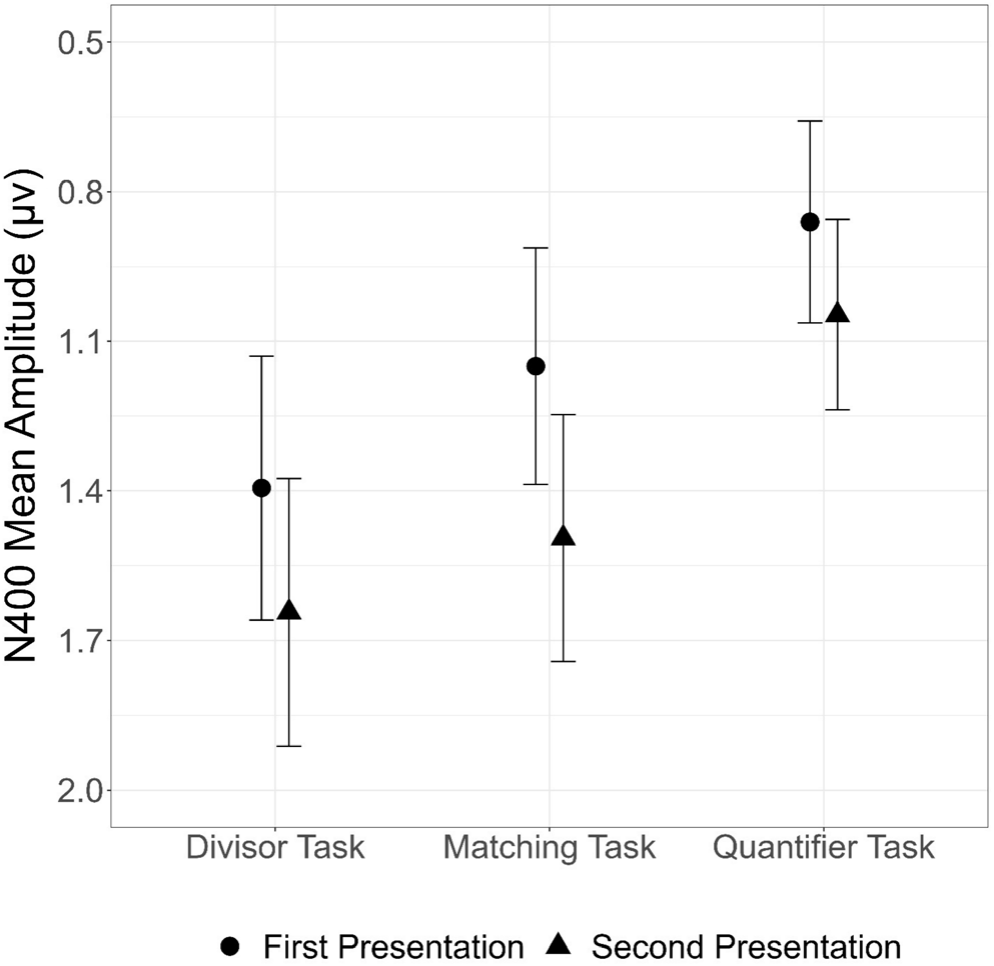
N400 mean amplitudes from 350 to 450 ms across all electrodes in the region of interest are graphed for first (circles) and second (triangles) presentations in each task type. Error bars give standard errors. Note that the y scale is reversed to remain consistent with the negative‐up plots of the ERP waveforms. N400 repetition effects were of similar size across the three tasks.

An ANOVA with factors of repetition (first and second presentations) and task (Matching, Divisor, and Quantifier) again revealed the main effect of repetition already described above (*F*(1, 29) = 10.17, *p* = 0.003), with larger N400 responses to first (0.39 μV) than to second (0.81 μV) presentations. There was no main effect of task (*F*(2, 58) = 1.81, *p* = 0.173) and no interaction between task and repetition (*F*(2, 58) = 0.374, *p* = 0.690). Figure 2 shows ERPs in each task; effect patterns are graphed in Figure 3.

#### 2.3.2 N400 Word Repetition Effects

To compare the repetition effects obtained for numbers with those for words, which are much better characterized in the literature, we also examined N400 responses to the word probes in the quantifier task as a function of repetition. We first assessed effects between 350 and 450 ms over medial centro‐posterior sites where word N400 effects are known to be generally largest (LDCe, LMCe, MiCe, RMCe, RDCe, LDPa, MiPa, RDPa, LMOc, MiOc, RMOc; Kutas and Federmeier 2011). A paired t‐test revealed a main effect of repetition (first versus second presentations), *t*(329) = 9.05, *p* < 0.001, with new items (0.69 μV) eliciting more negative N400 amplitudes than old items (1.65 μV).

Next, to compare the repetition effects between numbers and words, we computed a difference wave (second presentation minus first presentation) and conducted a distributional analysis using an ANOVA with two levels of stimulus type (words versus numbers), two levels of hemisphere (left and right), two levels of laterality (lateral and medial), and four levels of anteriority (prefrontal, frontal, central, and occipital). There was no main effect of stimulus type (*F*(1, 29) = 0.12, *p* = 0.729), indicating no substantial difference in the overall amplitude of the repetition effect across stimulus type. Stimulus type did not interact with hemisphere (*F*(1, 29) = 2.73, *p* = 0.109) or laterality (*F*(1, 29) = 0.018, *p* = 0.894) but did interact with anteriority (*F*(3, 87) = 14.82, *p* < 0.001). None of the higher‐order interactions between stimulus type and distributional factors were significant^4^.

**FIGURE 4:**
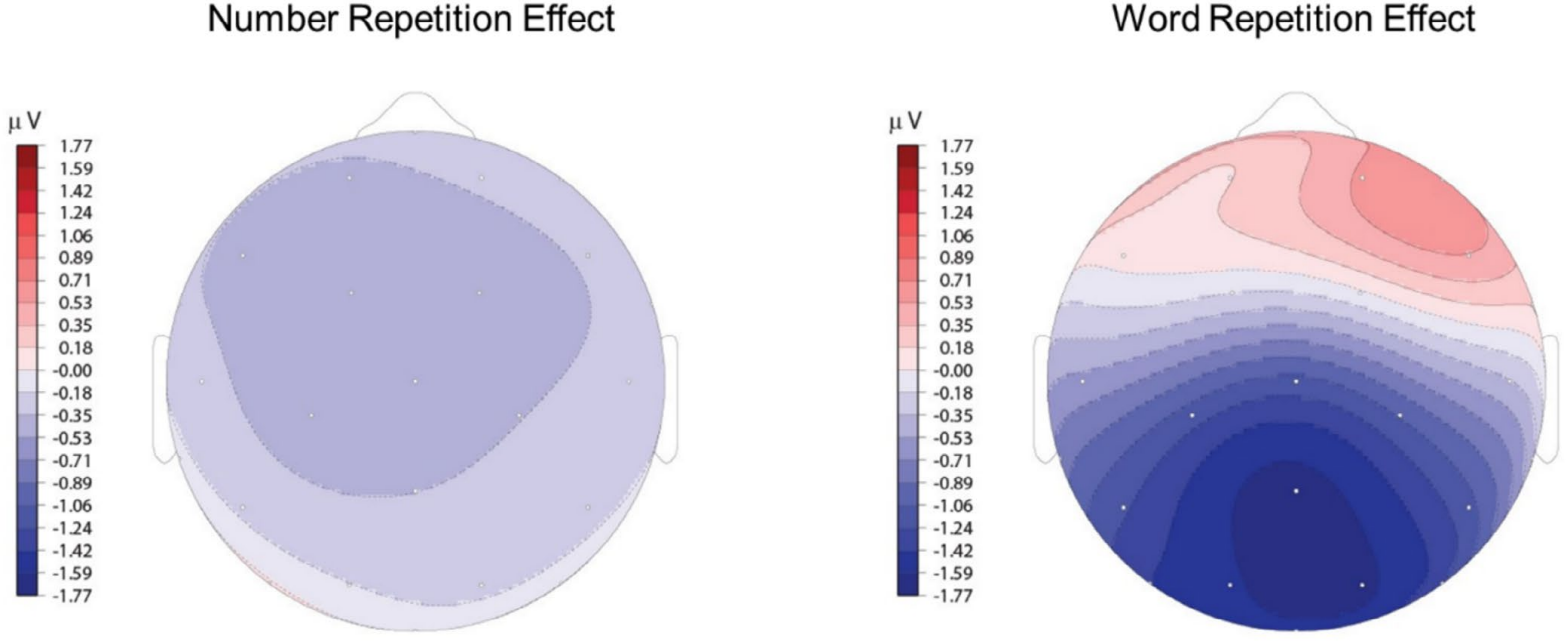
Topographical maps show the N400 repetition effect (first minus second presentation) distribution in the 350–450 ms time window for numbers (left) and words (right). As in prior work, repetition effects for words are largest over medial, centro‐posterior channels. In contrast, repetition effects for numbers have a fronto‐central maximum.

Pairwise *t*‐tests with Bonferroni correction indicated that whereas the repetition effect for numbers was larger at frontal sites compared to central and occipital sites (all *t*'s > 2.92), the repetition effect for words was larger over occipital sites (1.24 μV) compared to prefrontal (−0.48 μV), frontal (−0.02 μV), and central (0.65 μV) sites (all *t*'s > 5.55). Figure 4 shows the scalp distribution for number and word repetition effects.

## 3 Discussion

Although representations of numeric information may involve different cortical regions compared to representations of word meaning information (Ansari et al. 2006; Binder et al. 2009; Dehaene et al. 1999; Friederici et al. 2017), semantic access processes for a wide variety of stimulus types have been associated with the same kind of brain activity, reflected in the N400 ERP component (Kutas and Federmeier 2011). However, the question of whether Arabic numbers elicit N400 effects has been controversial. On the one hand, when people are prompted to connect double‐digit numbers with meaningful personal experience, repetitions of those numbers elicit N400 effects (amplitude reductions) that are similar to repetition effects for visual words in both timing and scalp distribution (Dickson and Federmeier 2018). On the other hand, in adults, numbers that constitute correct versus incorrect answers in the context of simple written arithmetic problems elicit P3b effects, rather than the kind of N400 effects that are observed for congruent versus anomalous words at the end of sentences (Grenier et al. 2020; cf., Dickson et al. 2018; Kutas and Hillyard 1984). This raises the question of whether numbers, when processed for their numerosity, access semantics differently from other types of stimuli.

To address this question, we examined repetition effects for numbers in three tasks wherein the repeating numbers were always presented before participants saw and responded to the task‐related probe items. The numbers repeated after a few trials, and those repetitions were incidental to the task. Because the numbers came before the probes, they could not act as targets and thus would be unlikely to elicit a P3b. In the Matching task, participants needed to retain a two‐digit number briefly in memory to be able to verify whether a subsequent single‐digit number was one of the initial digits. Succeeding in this task, therefore, did not require access to mathematical or numerosity associations with the numbers. The Divisor task was developed to tap into the kind of knowledge used in much prior work looking at N400 effects in the context of mathematics, but without the numbers serving as target answers. Following each two‐digit number, participants were given a single‐digit probe and asked to indicate if that probe was a factor of the original number. This task thus required participants to link numbers to their learned mathematical knowledge. Finally, in the Quantifier task, the numbers were followed by quantity words and participants indicated if they thought the numbers constituted common quantities for those terms. This task induced participants to access numerosity information without performing mathematical operations and without requiring the use of visual dot arrays, a popular technique for eliciting and studying numerosity processing, but one that can present challenging perceptual confounds for interpretation, especially in ERPs (Wilkey and Ansari 2020).

If the access of numerosity and related mathematical information from numbers is similar to other kinds of semantic access, we should see repetition effects as reductions in N400 amplitude between first and second presentations (Voss and Paller 2006; Voss et al. 2010; Voss and Federmeier 2011). Indeed, the results revealed reliably reduced negativity for second presentations of numbers compared to first between 350 and 450 ms. Moreover, the N400 repetition effect was of similar magnitude across all three tasks.

The presence of N400 repetition effects in the Quantifier task, which was designed to promote access to numerosity information, supports the idea that Arabic numbers can be linked to representations of quantity using processes that are similar to those involved in access to general semantics from numbers (as in Dickson and Federmeier 2018) and from other kinds of representations (e.g., words, pictures). Importantly, the same pattern was observed in the Divisor task, where the numbers were processed in the context of arithmetic problems. As discussed, prior work measuring ERPs in response to the presentation of the numerical solution (congruent or incongruent) of written arithmetic problems has yielded effects with conflicting interpretations. Initial studies interpreted the congruency‐based effect pattern as an N400‐based difference (Niedeggen and Rösler 1999; Niedeggen et al. 1999). However, more recent work has shown that this effect is likely driven by a P3b response to the correct target (Dickson et al. 2018; Grenier et al. 2020). Although adults can elicit N400 congruency effects to arithmetic problems under some conditions, such as when those problems are spoken (and, hence, when solutions are being processed as words; Dickson et al. 2018), in the context of well‐practiced written arithmetic problems, Arabic numbers may be processed as memorized sequences without undergoing much semantic analysis. In fact, there seems to be a developmental change in how written arithmetic expressions are processed since children do elicit N400 congruency effects to Arabic numerals in the context of simple arithmetic expressions (Grenier et al. 2020). Young children may be using a different processing strategy that accesses the math facts—perhaps because the problems are not yet well‐practiced. Importantly, in this study we also observed an N400 repetition effect in the Divisor task in our adult participants. This shows that adults also process written numbers for their numerosity information, even when the task demands are centered on arithmetic facts, as long as numbers are not presented as part of an over‐rehearsed sequence.

The observed repetition effects are particularly interesting in the case of the matching task, wherein participants were only required to confirm whether a probe digit was present in a double‐digit number. We expected the task to require very little access to numerosity information since it is possible to perform the task by matching the physical forms of the numbers. Nevertheless, we observed a similarly sized N400 repetition effect in this task, suggesting that people access numerosity information from Arabic numbers even when the task demands do not require them to do so. Accessing this kind of information by default may be an important part of how numbers are encoded to support later memory retrieval. For example, studies with the Pirahã, who speak a language that does not have terms denoting specific numerosities, have found that this language difference has an impact on the ability to remember previously encountered quantities (Gordon 2004; Frank et al. 2008). Encoding specific numeric information may be cognitively adaptive if one expects that the information will later need to be retrieved and manipulated.

The N400 repetition effect for numbers obtained across the three tasks had a different topographical distribution from that obtained for words in the same participants. N400 repetition effects for visually presented words have a typical centro‐posterior distribution that has been well‐characterized (Kutas and Federmeier 2011), which was also replicated here. In contrast, the N400 repetition effect for numbers in this study had a medial‐frontal distribution. Other types of stimuli, such as auditory words (Holcomb and Neville 1990; McCallum et al. 1984), pictures (Federmeier and Kutas 2002; Ganis et al. 1996), and faces (Olivares et al. 1999) have also been found to have different N400 distributions compared to visual words, and these differences have been interpreted as reflecting differences in the type of information being accessed from these stimuli, across a distributed semantic network (Manfredi et al. 2018; West and Holcomb 2002; cf., Khateb et al. 2010). Interestingly, although we did not test this statistically, the N400 topography observed in this study was also different from that observed to numbers when those numbers were being processed, not for their numerosity, but as cues for other types of world knowledge (Dickson and Federmeier 2018). In the latter case, the scalp distribution of the N400 repetition effect was very similar to that typically observed for visual words. Thus, it seems likely that the topographic difference observed here is not based on stimulus type as such (e.g., words vs. Arabic numbers), but on the type of knowledge that is being accessed from the stimuli (e.g., 711 meaning a particular quantity vs. 711 meaning “the convenience store where I buy my sandwiches”). Access to numerosity information may thus involve similar processing for other types of stimuli as part of a distributed semantic network, reflected in the N400 repetition effect pattern, but the information being activated—and thus shaping the scalp topography of the effect—is coming from different brain areas. Given that our study was not originally designed to compare N400 distributions across words and numbers or between numbers used to access numerosity versus more general semantic features, it will be important for future work to probe these distributional effects in a more targeted fashion.

In turn, the distributional differences we observe are consistent with work delineating different brain networks associated with the processing of numbers (Ansari et al. 2006; Dehaene et al. 1999) compared to those that have been linked to the processing of word semantics (Binder et al. 2009; Friederici et al. 2017). One brain area that has been particularly associated with the processing of numerosity is the intraparietal sulcus. Single‐cell recordings in monkeys have identified neurons in the lateral intraparietal area that are tuned to specific numerosities (Nieder and Miller 2004), and high‐resolution fMRI in humans has shown a numerosity‐based topographic mapping pattern in the intraparietal sulcus (Harvey et al. 2013). Although we do not know which brain areas are responsible for the repetition effects to numerals that we observed here, the topographic differences we observed are consistent with the idea that numerosity information, compared to general semantics, may be accessed from different brain areas, even when the eliciting stimulus in both cases is an Arabic numeral. At the same time, however, our results also suggest that numerosity information is not disconnected from the larger semantic system, in that it is engaged using the same mechanisms (thus yielding an N400 effect). Taken together, this suggests a means by which the semantic system can both develop processing for specialized purposes (as in the representation of precise quantity), while still allowing for connections across different representational types, so that, for example, those quantities can be linked with particular objects and their (other) semantic features.

In conclusion, across three tasks with differing task demands (accessing learned real‐word quantities, solving arithmetic problems, matching digits), we observed consistent N400 repetition effects for Arabic numbers. The insensitivity of the repetition effects to different task types indicates that, when Arabic numbers are processed for their numeric information, their semantic access is not strongly affected by task demands. Indeed, it may be that access to numerosity information from numbers is relatively automatic, similar to findings for words and pictures (Kutas and Federmeier 2011). At the same time, distributional differences between the repetition effect for numbers and words suggest that the representation of numerosity information and more general semantic information involves different brain networks.

## Author Contributions

**Will Deng:** data curation, formal analysis, visualization, writing – original draft, Writing – review and editing. **Danielle S. Dickson:** conceptualization, methodology, formal analysis, investigation, writing – original draft, writing – review and editing. **Kara D. Federmeier:** conceptualization, methodology, resources, supervision, project administration, writing – review and editing.

## Conflicts of Interest

The authors declare no conflicts of interest.

## Data Availability

The data that support the findings of this study are openly available in Open Science Framework at https://osf.io/9kjnq/?view_only=a320e644f63e4d2ca6d3f55a96907349.

## Endnotes

^1^
Children do elicit N400‐like responses in the context of simple math problems (Grenier et al. 2020).

^2^
The default position of the sliding ruler was placed at 50 (and to be registered as a response, participants would have to move it deliberately off of this position), so this bias towards being comfortable counting smaller numbers was not because the slider would need to be dragged farther to reach a higher number.

^3^
Repetition × hemisphere × laterality (*F*(1,29) = 0.13; *p* = 0.720); repetition × hemisphere × anteriority (*F*(3,87) = 1.63; *p* = 0.188); repetition × laterality × anteriority (*F*(3,87) = 1.66; *p* = 0.182); repetition × hemisphere × laterality × anteriority (*F*(3, 87) = 1.57; *p* = 0.202).

^4^
Stimulus type × hemisphere × laterality (*F*(1, 29) = 0.92, *p* = 0.346); stimulus type × hemisphere × anteriority (*F*(3, 87) = 0.32, *p* = 0.810); stimulus type × laterality × anteriority (*F*(3, 87) = 0.63, *p* = 0.597); stimulus type × hemisphere × laterality × anteriority (*F*(3, 87) = 2.55, *p* = 0.061).
